# The effects of hip- vs. knee-dominant hamstring exercise on biceps femoris morphology, strength, and sprint performance: a randomized intervention trial protocol

**DOI:** 10.1186/s13102-023-00680-w

**Published:** 2023-06-26

**Authors:** Scott K. Crawford, Jack Hickey, Jessica Vlisides, Jennifer S. Chambers, Samuel J. Mosiman, Bryan C. Heiderscheit

**Affiliations:** 1 Department of Kinesiology, University of Wisconsin-Madison, Madison, WI USA; 2 Department of Orthopedics and Rehabilitation, University of Wisconsin-Madison, Madison, WI USA; 3 School of Behavioural and Health Sciences, Australian Catholic University, Fitzroy, VIC Australia; 4 Sports Performance, Recovery, Injury and New Technologies Research Centre, Australian Catholic University, Fitzroy, VIC Australia; 5 Department of Sport Science and Nutrition, Maynooth University, County Kildare, Ireland; 6 Badger Athletic Performance Program, University of Wisconsin-Madison, Madison, WI USA; 7 Department of Kinesiology, Department of Orthopedics and Rehabilitation, University of Wisconsin-Madison, 1300 University Ave, Madison, WI 53706 USA

**Keywords:** Eccentric strength, Resistance training, Hamstring muscles, Injury prevention, Sprint running

## Abstract

**Background:**

The hamstrings are an important muscle group that contribute to horizontal force during sprint acceleration and are also the most injured muscle group in running-based sports. Given the significant time loss associated with hamstrings injury and impaired sprinting performance following return to sport, identifying exercises that drive adaptations that are both protective of strain injury and beneficial to sprint performance is important for the strength and conditioning professional. This paper describes the study protocol investigating the effects of a 6-week training program using either the hip-dominant Romanian deadlift (RDL) or the knee-dominant Nordic hamstring exercise (NHE) on hamstring strain injury risk factors and sprint performance.

**Methods:**

A permuted block randomized (1:1 allocation) intervention trial will be conducted involving young, physically-active men and women. A target sample size of 32 will be recruited and enrolled participants will undergo baseline testing involving extended-field-of-view ultrasound imaging and shear wave elastography of the biceps femoris long head muscle, maximal hamstrings strength testing in both the RDL and NHE, and on-field sprint performance and biomechanics. Participants will complete the 6-week training intervention using either the RDL or NHE, according to group allocation. Baseline testing will be repeated at the end of the 6-week intervention followed by 2 weeks of detraining and a final testing session. The primary outcome will be regional changes in fascicle length with secondary outcomes including pennation angle, muscle cross sectional area, hamstring strength, and maximal sprint performance and biomechanics. An exploratory aim will determine changes in shear wave velocity.

**Discussion:**

Despite extensive research showing the benefits of the NHE on reducing hamstring strain injury risk, alternative exercises, such as the RDL, may offer similar or potentially even greater benefits. The findings of this study will aim to inform future researchers and practitioners investigating alternatives to the NHE, such as the RDL, in terms of their effectiveness in reducing rates of hamstring strain injury in larger scale prospective intervention studies.

**Trial Registration:**

The trial is prospectively registered on ClinicalTrials.gov (NCT05455346; July 15, 2022).

**Supplementary Information:**

The online version contains supplementary material available at 10.1186/s13102-023-00680-w.

## Background

The hamstrings muscles act to both extend the hip and flex the knee, which are particularly important movements during sprinting. The hamstrings are primary contributors to horizontal force production during both acceleration and maximal velocity phases of sprinting [1, 2] with their contribution to propulsion increasing substantially as an athlete nears maximum speed [3]. Due to the increased lengthening and high negative work done by the hamstrings during the swing phase of sprinting [4–6], the hamstrings are also highly susceptible to injury in running-based sports [7–9]. Considering the significant incidence and subsequent time loss of hamstring strain injuries (HSI) in sport [8–14], identifying exercises that emphasize adaptations that are both protective of strain injury and beneficial to sprint performance is important.

One exercise that has garnered a lot of attention as an effective component for HSI prevention is the Nordic hamstring exercise (NHE), which involves minimal equipment and has been integrated within injury prevention programs, such as the FIFA11+ [15, 16]. The high eccentric demand placed on the hamstrings during the exercise [17] is a potent stimulus for inducing beneficial adaptations thought to be protective of HSI. These adaptations include increased fascicle length with concurrent decreases in pennation angle, muscle hypertrophy, and increased eccentric knee flexor strength [18–21]. Additional ultrasound-derived shear wave speed (SWS)—a proxy for muscle material properties and stiffness—may also contribute to injury resiliency [22], though chronic adaptations following eccentric training have not been consistently established [23].

Studies using both surface electromyography (sEMG) and magnetic resonance imaging have shown the NHE preferentially recruits the semitendinosus compared to the biceps femoris—the latter of which is the more frequently injured hamstrings muscle [17, 24, 25]. One possible explanation for the preferential recruitment of the semitendinosus is that the NHE is a knee-dominant exercise, whereas studies have shown the biceps femoris muscle is more active (proportionally to the semitendinosus muscle) in hip dominant exercises, such as the 45-degree hip extension and Romanian deadlift (RDL) [17, 24, 26–29].

The NHE loads the hamstrings at relatively short muscle lengths [30–33] compared to the RDL, the latter of which can be progressively loaded throughout a greater range of motion [34, 35]. Higher neuromuscular activation was also observed in the proximal region compared to the distal region of the biceps femoris muscle during the stiff-leg deadlift – a similar exercise to the RDL [36]. Together, the higher lateral to medial hamstrings muscular activation ratio and force generation throughout longer muscle lengths induced by the RDL may increase the potential to elicit potentially beneficial adaptations in the injury-susceptible biceps femoris muscle [1–6].

Compared to its injury prevention effects, the impact of the NHE on sprint performance is less clear [37–41]. The implications of the RDL as it relates to improving sprint performance also have not been well-described. Sprint performance is often defined by time, but this measure provides limited information into different aspects (e.g., sprint mechanics) or phases (e.g., acceleration, transition, maximal velocity) of a sprint. Kinematics are often assessed during maximal velocity sprinting and relate to both performance and hamstrings injury susceptibility [42, 43], suggesting the importance of assessing sprint performance with other metrics than just total or split times. Simple biomechanical methods (i.e., force-velocity profiling) have been described to assess sprint kinetics during a 60 m sprint [44]. These methods may be useful in differentiating between horizontal force and maximal velocity contributions to sprint performance [2, 44–47].

Considering the hamstrings’ contribution toward horizontal force production during sprinting, the anatomical determinants that may predispose the biceps femoris muscle toward force production, and the significant stretch the biceps femoris undergoes during swing phase [1, 2, 4, 48, 49], selecting exercises that target this muscle, particularly in an eccentrically-biased manner, could prove vital for muscular adaptations for both injury risk mitigation and sprint performance. Yet, it is unclear how eccentric training, particularly with RDLs, influences sprint performance. Additionally, architectural adaptations to training interventions should be investigated along the length of the muscle to determine if changes are more pronounced in different regions [48, 50] and if these correspond to regions more susceptible to strain injury [51], such as the proximal muscle-tendon junction of the biceps femoris [52, 53].

Therefore, the purpose of the study is to investigate the effects of a 6-week training program matching volume and eccentric contraction time between the RDL and the NHE on hamstrings architecture, hamstrings strength, and sprint performance. As an exploratory aim, ultrasound shear wave speed (SWS) will also be measured along the length of the muscle to determine chronic adaptations in tissue material properties following eccentric training. Findings from this study will aim to provide evidence for targeted exercise selection in addressing HSI risk factors with potential to inform rehabilitation programming.

## Methods/Design

### Overall study design

This study is a randomized intervention trial where participants will be allocated to one of two experimental groups: RDL or NHE training. The primary outcome of the study is the change in regional biceps femoris fascicle length between the two intervention groups. Secondary outcomes will include ultrasound-derived changes in pennation angle, muscle thickness, anatomical cross-sectional area, in addition to hamstrings strength, and maximal sprint performance. As an exploratory aim, we will determine changes in regional SWS following training and between the two intervention groups.

The study is registered on ClinicalTrials.gov (Identifier: NCT05455346) with all procedures approved by the Health Sciences Institutional Review Board at the University of Wisconsin-Madison. Written informed consent will be obtained from all participants before inclusion by study personnel with Human Subjects Protection training. Any changes in the protocol will be reflected on the clinical registration website of ClinicalTrials.gov.

**Fig. 1:**
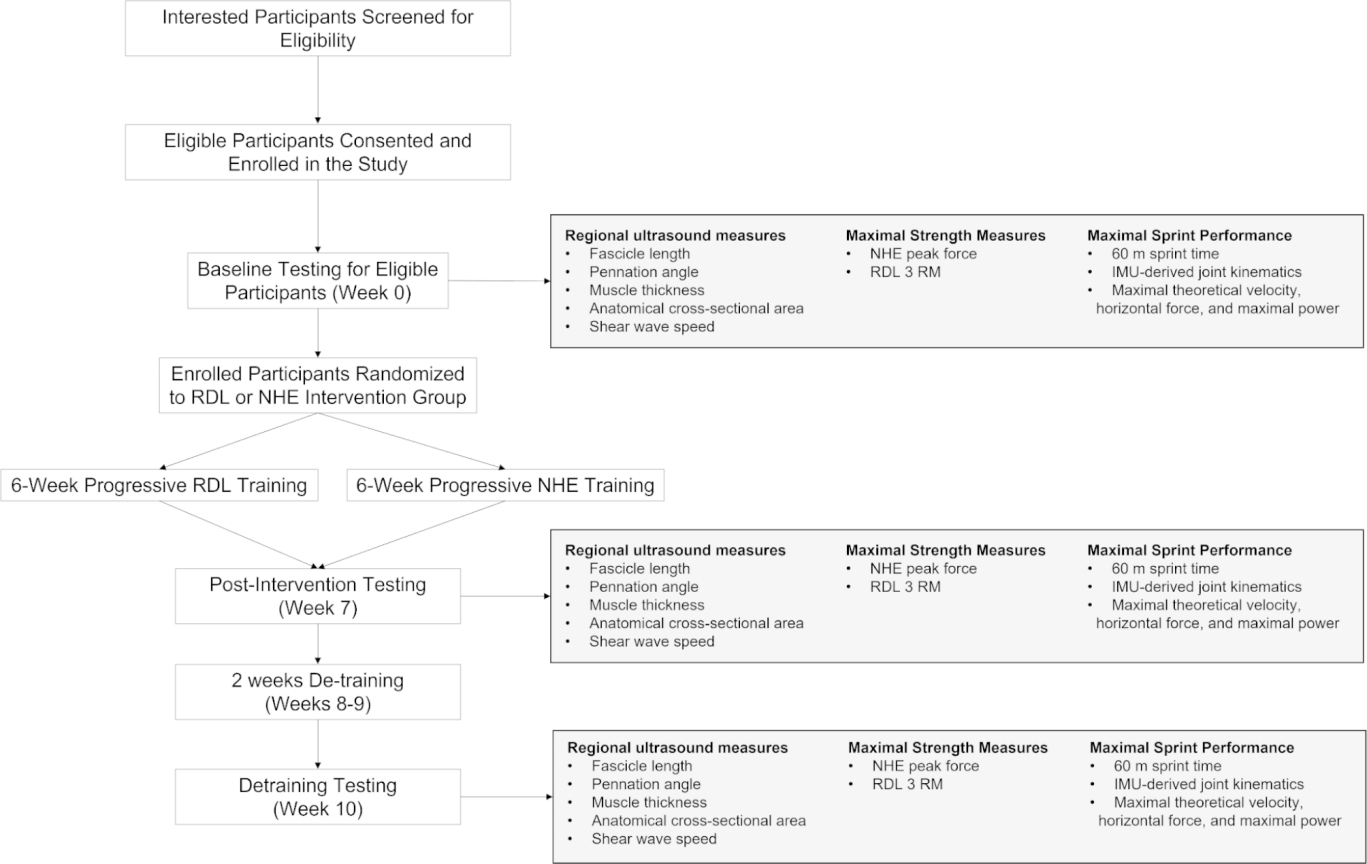
Proposed experimental design and outcome measures at each testing time-point

An overview of the experimental design is given in Fig. 1. Inclusion criteria for participants eligible for the intervention trial are 18–25 years of age (consistent with previous investigations [29, 47, 54]), self-report of being physically active—as defined by the Physical Activity Guidelines for Americans [55],, having > 6 months experience in resistance training, no history of HSI within the last 6 months, no history of lower extremity surgery, no current musculoskeletal injury to the lower extremity, and females not currently pregnant. Physical activity will be defined as participating in weekly totals of 150–300 min (5 h) of moderate-intensity, or 75–150 min (2 h and 30 min) of vigorous-intensity aerobic physical activity, or an equivalent combination of moderate- and vigorous-intensity aerobic activity. Prior to the intervention, baseline assessments will be performed. These include regional ultrasound measures (see *Ultrasound Measures*); strength for the NHE and RDL (see *Nordic Hamstring Exercise and Romanian Deadlift Strength Testing*); and maximal 60 m sprints (see *Maximum Sprint Testing*). Each participant will also be asked to complete the Baecke questionnaire for determination of habitual physical activities and what current physical activities pertinent to the study in which they participate at baseline [56]. Participants will be able to continue usual levels and mode of physical activity but be asked to refrain from supplementary lower extremity resistance training and sprint-specific training.

An *a priori* power calculation using R statistical software and “WebPower” package (wp.kanova function) [57] was performed. Using an effect size based the median Cohen’s d extracted from several relevant studies investigating the effects of NHE on the primary aim of fascicle length changes [19, 21, 26, 54, 58] and converting to Cohen’s f (f = 0.8), 2 experimental groups (RDL vs. NHE), a total of 12 factors (levels of intervention x time x imaging location (proximal, mid-belly, distal) = 2 × 2 × 3), α = 0.05, and power = 0.80, a total sample size of 11 participants per group will be required. This sample size is consistent with previous investigations of fascicle length following NHE training [19, 21, 26, 54, 58]. Using a conservative effect size (Cohen’s d = 0.5) based on observed 2% differences detected in sprint times between two eccentric training interventions (Cohen’s d = 0.87) [47] and converting to Cohen’s f (f = 0.25), a total sample size of 28 (N = 14 per group) will be required for the secondary aims. Based on the larger sample size and accounting for a 10% attrition rate, a total of 16 participants per group (N = 32 in total) will be recruited from the University of Wisconsin to participate. Participants will be compensated on a pro-rated basis for completing study visits and provided their strength and sprint results.

Participants will be allocated to either the RDL or NHE training group using a random permuted block randomization (1:1 training group allocation) using *a priori* computer-generated group numbers. Each participant will undergo the 6-week intervention program based upon their group allocation (see *Intervention*). A 6-week period was chosen due to previous observations that additional changes in fascicle length (primary outcome) were minimal with NHE training lasting > 6 weeks [47, 59] and is consistent with previous investigations of architectural changes induced by NHE [54, 58]. Following the 6-week intervention, participants will return to the lab for post-intervention assessments of regional ultrasound of the hamstrings, RDL and NHE strength testing, and sprint testing, which will be carried out in the same manner as baseline assessments. Fascicle length changes are known to return to baseline values within 2 weeks after the cessation of NHE [54, 60]. To investigate the potential short-term washout effects of the training intervention on the primary and secondary outcomes, participants will be asked to return to the lab following a 2-week detraining period. During these 2 weeks, participants will be encouraged to resume all normal activities but limit any outside eccentric hamstring strength training.

### Ultrasound

Participants will lay prone on an exam table with their hips and knees in a neutral position and feet off the end of the exam table. Participants will lie quietly at rest for 3 min prior to image acquisition to normalized fluid shift within the muscle [61, 62].

Ultrasound B-mode images will be collected unilaterally from the biceps femoris muscle of the dominant limb for each participant using a Logiq P9 ultrasound system (GE Healthcare, Waukesha, WI) and a linear array transducer (L3-12-RS, 47.1 mm aperture). The same researcher (SKC) will perform all image acquisitions and will be blinded to participant group allocation. The thigh length from the ischial tuberosity to the midpoint between the femoral condyles will be measured and recorded. Skin marks will be made at 33%, 50%, and 67% of the thigh length from the ischial tuberosity to standardize imaging locations between participants and correspond to approximately proximal, mid-belly, and distal regions of the hamstring muscle, respectively [63, 64].

**Fig. 2:**
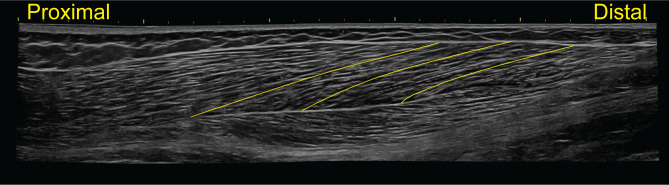
Representative longitudinal extended-field-of-view image of the biceps femoris long head muscle with representative fascicles in the proximal, mid-belly, and distal regions (from left to right) outlined and highlighted in yellow

Three longitudinal extended-field-of-view images of the entire biceps femoris muscle (i.e., from the most proximal to the most distal visualization of the muscle-tendon junctions) will be collected. According to extended-field-of-view image acquisition recommendations and previous studies [65–71], preliminary scans will be performed to determine the proximal and distal muscle-tendon junctions A longitudinal image will be captured along the path of the fascicle plane spanning the entire length of the biceps femoris muscle (Fig. 2).

**Fig. 3:**
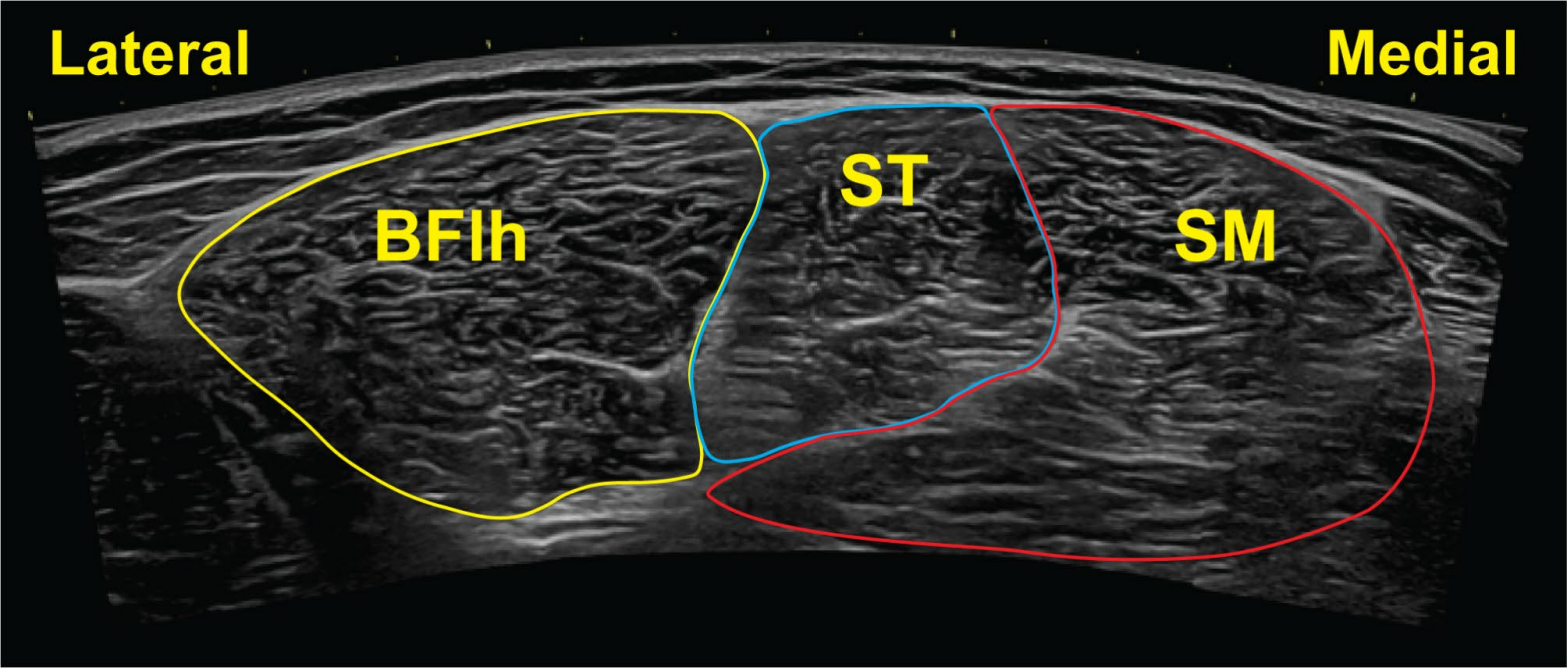
Representative transverse extended-field-of-view image of the biceps femoris long head muscle (BFlh), semitendinosus (ST), and semimembranosus (SM) muscles. Anatomical cross-sectional area will be calculated from the transverse ultrasound images

Three transverse extended-field-of-view images will be captured at the proximal, mid-belly, and distal locations to determine regional anatomical cross-sectional area of the biceps femoris long head muscle. The transducer will be placed perpendicular to the skin and the entire cross-sectional view of the hamstrings muscles imaged (Fig. 3) [70, 72]. A transverse image using traditional field-of-view B-mode imaging will also be captured at these locations.

**Fig. 4:**
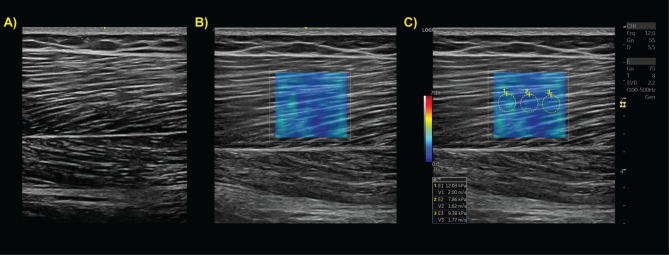
Representative ultrasound (A) B-mode image of the mid-belly biceps femoris long head muscle, (B) shear wave map at the same location, and (C) Q-box measures within the shear wave map with corresponding values in bottom lefthand subpanel

To determine changes in tissue stiffness characterized by ultrasound SWS for the exploratory aim, the same transducer will be placed in the same proximal, mid-belly, and distal locations in a longitudinal view. A traditional field-of-view B-mode image will also be captured at these locations. This orientation (parallel to the muscle fascicles) has been shown to be more reliable for measuring SWS than transverse views [73, 74]. Minimal pressure will be applied to the muscle and the shear wave box (for wave speed detection) will be placed in the middle of the imaged muscle region (Fig. 4). Shear wave maps will be generated by the ultrasound system and SWS measures will be extracted from the middle of the SWS map using the measurement tools of the ultrasound system.

### Nordic hamstring exercise and romanian deadlift strength testing

Hamstrings strength testing will be determined for 3 repetitions of the NHE and three-repetition maximum (3RM) in the RDL following ultrasound imaging. Prior to each test, participants will perform a standardized 5-minute general warm-up on a stationary bike followed by a task-specific warm-up including 3 sets of submaximal trials of the NHE and RDL (Additional File 1, Table A1). The RDL testing will precede the NHE for all participants with a full 15-minute rest between exercises to minimize fatigue.

The RDL will be performed in a multi-purpose, commercial power rack with a standard 20 kg Olympic barbell. Incremental load increases (5–20%) will be added for each subsequent trial until the participant cannot complete 3 repetitions through the full range of motion using proper technique. Participants will use wrist straps during the 3RM determination so maximal RDL loads will not be influenced by the participant’s grip strength. A 3 min rest will be administered between all warm-up and testing sets to allow for full recovery.

The NHE will be performed on the Nordbord (Vald Performance, Queensland, Australia), which is a reliable device to assess maximum eccentric knee flexor strength [75]. NHE testing will be performed consistent with previous investigations [2, 47, 54, 59, 60]. Participants will begin kneeled on the device in approximately 90° of flexion while their ankles are secured into fixed hooks placed superior to the lateral malleoli and oriented vertically. Participants will be asked to maintain a dorsiflexed ankle when performing the NHE [76]. Participants will cross their arms over their chest and be instructed to maintain a neutral (0° extension) hip posture while lowering their upper body to the ground as slowly as possible. They will be instructed to uncross their arms and gently catch themselves before hitting the ground if they feel their hips alignment “break” (i.e., they are unable to maintain 0° hip alignment).

Following submaximal NHE practice trials, participants will begin the maximal test, which will consist of 1 set of 3 maximal repetitions. Minimal rest (< 2 s) will be permitted between reps. If participants can control the eccentric portion of the movement towards full knee extension (i.e., not falling beyond ~ 20° knee flexion), then participants will repeat a subsequent set of 3 maximal repetitions while holding external load (5 kg increment) positioned on sternum. In the event participants need to sequentially add external load for maximum NHE testing, full recovery (3 min) will be administered between sets.

### Maximum sprint testing data collection

A minimum of 15 min between strength testing and sprints will be administered. Sprints will be performed on artificial turf to minimize any effects of weather and variations in ground surface conditions on sprint performance outcomes. Prior to sprinting, participants will be weighed for subsequent sprint kinetics analysis (see *Outcome Measures & Statistical Analysis*) and to determine an accurate mass for proper calibration of the IMU system.

Participants will go through a standardized warm-up (Additional File, Table A2) [2, 77]. Following the warm-up, the participants will rest for 5 min to allow for placement of eight IMUs (Xsens MVN, Xsens Technologies B.V., The Netherlands) on the sternum, sacrum, and bilaterally on the thighs, legs, and foot. This system is reliable and validated to measure joint kinematics during dynamic trials and related to sprint mechanics [78–80]. Once the system is successfully calibrated, participants will then perform three maximal 60 m sprints.

Timing gates (SmartSpeed, Vald Performance, Queensland, Australia) will be placed at the start line (0 m), 5, 10, 20, 30, 40, 50, and 60 m. Each participant will perform a total of 3 maximal 60 m sprints from a standing position with 90 s rest in between each trial to minimize the effects of fatigue. Participants will begin with their front foot 0.5 m behind the first timing gate to prevent premature activation of the timing gates.

### Intervention

All training sessions will be supervised by key study personnel with more than 4 years of exercise testing and prescription to monitor the training sessions and to keep the assessor for the primary outcome (i.e., fascicle length) blinded to the group allocation of individual participants. The key study personnel responsible for implementing the training intervention will contact each participant weekly for training sessions. A metronome (60 Hz) will be used to provide feedback for the execution of the eccentric contraction time throughout the range of motion.

Prior to each training session, participants will undergo a standardized warm-up that will include a 5 min general warm-up on a stationary bike followed by specific dynamic drills to prepare the athlete for the training session (Additional File, Table A1). The training intervention groups will undergo the same program with the only difference being the interventional hamstring exercise. The program will consist of a 2-week familiarization period followed by 4-weeks of progressive training [54]. A sample of the overall resistance training program is shown in Table 1 with each training session expected to last 45–60 min.

**Table 1:** Overview of the entire 6-week resistance training program

*Exercise*	*Sets x Repetitions (ECC:ISO:CON tempo)*	*Frequency* *(sessions per week)*	*Total weekly repetitions*	*Intensity*	*Inter-set Rest (sec)*
2-week Familiarization Period					
Hamstring exercise	As per intervention group allocation				
Incline DB Press	2 × 10 (2:0:1)	2	40	70–75% 1RM (7 RPE)	60
Chest-supported Incline DB Row	2 × 10 (2:0:1)	2	40	70–75% 1RM (7 RPE)	60
Prone Shoulder Y-T-W	2 × 10 (2:0:1)	2	40	70–75% 1RM (7 RPE)	60
Biceps curls to Triceps pressdown	2 × 15 (2:0:1)	2	60	70–75% 1RM (7 RPE)	60
Half Ups-Half Downs Crunches	2 × 10 each (1:1:1)	2	40	Bodyweight	60
4-week Progressive Training Period					
Hamstring exercise	As per intervention group allocation				
Incline DB Press	3 × 10 (2:0:1)	2	60	70–75% 1RM (7 RPE)	60
Chest-supported Incline DB Row	3 × 10 (2:0:1)	2	60	70–75% 1RM (7 RPE)	60
Prone Shoulder Y-T-W	3 × 10 (2:0:1)	2	60	70–75% 1RM (7 RPE)	60
Biceps curls to Triceps pressdown	3 × 12 (2:0:1)	2	72	70–75% 1RM (7 RPE)	60
Half Ups-Half Downs Crunches	3 × 10 each (1:1:1)	2	60	Bodyweight	60

Abbreviations: DB = dumbbell, RM = repetition maximum, RPE = rate of perceived exertion, ECC = eccentric, ISO = isometric, CON = concentric

The hamstring-specific training will consist of the same number of repetitions performed between the RDL and the NHE. The NHE will be performed in the power rack with a pad placed under the knees and the ankles secured using the safety pins. Participants will be instructed to maintain their ankles in a dorsiflexed position, lower themselves as close to the ground as possible at a constant and controlled speed while maintaining a neutral position of the hips and trunk, and to cross their arms in front of their chest. Once an athlete reaches the ground, they will push themselves back up to the starting position while minimizing the amount of time between repetitions (< 2 s). During the familiarization period, the relative intensity will be lower compared to the 4-week progressive training to ensure each participant performs the exercise with proper technique and progressively works up to the demands of the intervention. We have observed in testing that most athletes complete the NHE in approximately 4 to 6 s [81]. This is consistent with a recent study in male field hockey players where mean (standard deviation) time to complete the NHE was 4.17 (1.14) seconds with a dorsiflexed ankle [76]. The relative intensity will be progressively increased by modifying the time to complete the NHE (from 4 to 6 s, Table 2) throughout the training period. The relative intensity during the intervention (8–9 RPE) will be maintained across the NHE by having athletes hold weight plates across their chest (as necessary). Training logs will be used to track athletes’ training loads.

The RDL will be performed in a similar manner as the 3RM testing with the barbell placed on the safety pins positioned slightly below the knee. The time to complete the eccentric portion of the RDL will be performed in a progressive manner and time-matched with the NHE (Table 2) with the athlete returning to the start position with a maximal concentric hip extension. Relative training intensity will be maintained by adding or removing resistance to the exercise. The prescribed 8–9 RPE equates to approximately 82–92% 3RM. Wrist straps will be worn during the RDL training sessions to ensure the training loads adhere to the intensity prescription and are not limited by the participant’s grip strength. The description of the interventional program is shown in Table 2. Both groups will train a total of 2 sessions per week.

**Table 2:** Hamstring intervention program

*Training Period*	*Week*	*Frequency (sessions per week)*	*Sets x Reps*	*Target Eccentric Contraction Duration* *(sec per rep)*	*Target intensity* *(0 to 10 RPE)*	*Inter-set rest (sec)*
**Familiarization**	1	2	3 × 3	4 s through full ROM	5	90
	2	2	3 × 4	5 s through full ROM	7	90
**Intervention**	3	2	4 × 4	6 s through full ROM	≥ 8	120
	4	2	4 × 4		≥ 8	120
	5	2	4 × 4		≥ 8	120
	6	2	4 × 4		≥ 8	120

† Training prescription applied twice per week over the 6-week intervention period for both Nordic hamstring exercise (NHE) and Romanian deadlift (RDL) groups. NHE will be performed to each participant’s maximum range of motion at the prescribed eccentric tempo. RDLs will be executed at the prescribed tempo ranging between 4–6 s of eccentric lowering and returning to the start position as fast as possible. Absolute intensity progressively increased on an individual basis in both groups by adding external load to ensure target eccentric contraction duration and intensity is being met as prescribed below

Abbreviations: Rep(s) = repetition, ROM = range of motion, sec = seconds

### Image analysis

Ultrasound images will be extracted from the ultrasound machine and analyzed offline. Using publicly available software (ImageJ, National Institutes of Health), the fascicle length, pennation angle, and muscle thickness from the extended-field-of-view and static B-mode longitudinal images will be analyzed according to previous methods [68, 69]. Anatomical cross-sectional area will be calculated from transverse images at the proximal, mid-belly, and distal locations.

### Outcome measures & statistical analysis

All outcome results will be included in an anonymous database for statistical analysis. Using an intention-to-treat analysis, separate linear mixed effects models will be used to compare the effect of RDL and NHE on the primary outcome of biceps femoris long head fascicle length. Similar analyses will be performed for secondary analyses related to pennation angle, anatomical cross-sectional area, muscle thickness, and SWS. Full factorial models will be implemented with fixed effects of intervention group, muscle region, and time and a random effect to account for between-participant variation. Effect sizes will be calculated.

Sprint performance outcomes will be summarized using descriptive statistics. Given that the expected changes in sprint performance are numerically small but may still represent meaningful changes in overall sport performance and improvement, we will set *a priori* thresholds to describe the magnitude of change. Small, medium and large improvements in sprint times will be defined as < 2%, 2–4%, and > 4%, respectively, based off changes in 40 m sprint times observed previously [47, 82].

Discrete kinematic variables will be summarized using descriptive statistics and compared between time points for the maximal sprint using linear mixed effects models [83]. Due to the continuous time series data extracted from the IMUs, statistical parametric mapping (SPM) will also be used to determine any within-participant changes in kinematics throughout the entire sprint. SPM allows for the analysis of the entire time series and has been used in a variety of biomechanical studies [84–86]. Specifically, within-subject differences in trunk, hip, knee, and ankle kinematics will be calculated between test sessions

As an additional description of sprint performance, we will characterize the horizontal force production as detailed by Samozino et al. and adapted by others [1, 47, 87]. Descriptions of the theoretical maximal horizontal force (F_0_), velocity (v_0_), and maximal power output (P_max_) will be derived from participants’ split times and each participant’s body mass according to previous works [44, 88]. Intervention assessments for each variable across each time point will be compared using linear mixed effects models and R software [89, 90]

## Discussion

Although much research has been dedicated to identifying the etiology and mechanisms of injury, potential risk factors, residual neuromuscular deficits, and evidence-based recommendations for rehabilitation protocols of HSI [4, 6, 10, 13, 18, 91–102], the incidence and injury burden of HSI have not improved [14, 103]. The NHE is a staple in injury prevention programs for HSI, but evidence suggests adherence is a major limitation in its effectiveness for reducing HSI [104–107]. Due to the high rate of injury within the proximal muscle-tendon junction of the biceps femoris and non-uniform muscular adaptation to resistance training, different exercises should be employed to target specific muscle regions and increase eccentric training adherence [52, 53, 108, 109]

Consistent eccentric hamstring strength training (specifically using the NHE) increases eccentric knee flexor strength and influences architectural adaptations thought to be protective of HSI—such as increased fascicle length with concurrent decreases in pennation angle [47, 50, 54, 59, 60, 97, 110]. Fascicle length, pennation angle, and muscle thickness are typically measured using ultrasonography at the mid-belly [2, 47, 54, 59, 60], but this does not account for the known variation in architecture along the length of the hamstrings muscles [48, 49, 111, 112] or the non-uniform lengthening, activation, and adaptations induced by resistance training [113–116]. Recent evidence indicated that changes in fascicle and sarcomere lengths only occur in the distal region of the biceps femoris muscle following NHE training [50]. This coincides with higher neuromuscular activation in the distal region compared to the proximal and middle regions of the biceps femoris muscle during the NHE [117]. Our proposed investigation aims to determine if hip-dominant (RDL) or knee-dominant (NHE) eccentric hamstring exercises influence regional differences in fascicle length adaptations

Although ultrasonography is typically used to characterize architectural adaptations following eccentric exercise training, some ultrasound systems have capabilities that can provide insight into characterizing tissue material properties. Shear wave imaging has been shown to be a reliable, non-invasive, quantitative technique to characterize tissue stiffness in both tendon [118–120] and muscle [74, 121, 122]. Ultrasound elastography-derived shear modulus (derived from the SWS) has also been related to both passive and active muscular force production [123] as well as isometric rate of torque development in the gastrocnemius muscle [124]. Therefore, SWS may relate to both sprint performance and injury resiliency, but chronic adaptations following eccentric training have not been consistently established [23]. As an exploratory aim, we will measure regional SWS in the biceps femoris muscle to determine any chronic adaptations in tissue material properties following eccentric strength training

Resistance training, specifically eccentric training, has shown positive effects for both injury prevention and athletic performance. Despite extensive research showing the benefits of the NHE on reducing HSI risk [125], there is ongoing debate whether alternative exercises, such as the RDL, may offer similar or potentially even greater benefits. This is particularly debated with respect to the biceps femoris muscle, which is most frequently injured and appears to be more proportionally targeted in hip extension versus knee flexion exercises [17, 25, 26, 29, 52, 53]. While it is acknowledged that interventions aimed at hamstring injury prevention and improving sprint performance should be multi-faceted, comparing two commonly prescribed exercises like the NHE and RDL allows practitioners to prescribe hamstring exercises in an evidence-based manner with descriptions of the underlying muscular adaptations and implications for sprint performance improvements. The methods and proposed sprint analyses will allow for a comprehensive view of the effects of both the NHE and RDL on sprint mechanics through force-velocity profiling and kinematic changes throughout key phases of the sprint

The findings from this proposed study have practical applications for strength and conditioning coaches, athletic trainers, and physical therapists to address HSI risk factors and sprint performance through targeted exercise selections. In addition to providing information for evidence-based hamstring exercise selection, it is hoped that the findings of this study also inform future research projects aimed at investigating alternatives to the NHE, such as the RDL, in terms of their effectiveness in reducing rates of HSI in larger scale prospective intervention studies

## Electronic supplementary material

Below is the link to the electronic supplementary material.

Supplementary Material 1

Supplementary Material 2

Supplementary Material 3

Supplementary Material 4

## Data Availability

The anonymized dataset used for analysis will be made available from the corresponding author upon reasonable request.

## Declarations

### List of abbreviations

HSI = hamstring strain injury, IMU = inertial measurement unit, NHE = Nordic hamstring exercise, RDL = Romanian deadlift, SPM = statistical parametric mapping, SWS = shear wave speed.
