# Supplementary material for: The effects of hip- vs. knee-dominant hamstring exercise on biceps femoris morphology, strength, and sprint performance: a randomized intervention trial protocol
Source: BMC Sports Sci Med Rehabil. 2023 Jun 26;15:72. doi: 10.1186/s13102-023-00680-w (PMC10294505; doi:10.1186/s13102-023-00680-w)
Supplement: Supplementary file 2 — Supplementary Material 2 [file 13102_2023_680_MOESM2_ESM.docx]

**Additional File 3:**

**Table A2.** Sprint Testing Warm-Up Protocol

| Exercise | Warm-up Prescription |
| --- | --- |
| Jog/Run | 2 minutes at self-selected speed |
| Running pigeon | 6-8 each side |
| Adductor rock back | 6-8 each side |
| Ankle 3-way mobilization | 5 seconds in all directions |
| Hip mobilization & lumbar rotation | 6-8 each side |
| Grass mowers | 10yd forward and back |
| A-skips forward | 20yd |
| C-skips backward | 20yd |
| 1. Single-leg alternating bound for distance 2. High skips back | 10yd  10yd |
| Submaximal sprints | 50%, 70%, 90% |
