# Supplementary material for: The effects of hip- vs. knee-dominant hamstring exercise on biceps femoris morphology, strength, and sprint performance: a randomized intervention trial protocol
Source: BMC Sports Sci Med Rehabil. 2023 Jun 26;15:72. doi: 10.1186/s13102-023-00680-w (PMC10294505; doi:10.1186/s13102-023-00680-w)
Supplement: Supplementary file 3 — Supplementary Material 3 [file 13102_2023_680_MOESM3_ESM.docx]

**Additional File 1:**

**Table A1.** Strength Testing & Intervention Warm-Up Protocol

| Exercise | Warm-up Prescription |
| --- | --- |
| Stationary bike | 5 minutes at self-selected speed |
| Ankle 3-way mobilization | 5 seconds in all directions |
| Open book | 10 each side |
| Hip mobilization & lumbar rotation | 10 each side |
| Seated hip internal rotation mobilization | 8 each side |
| Runner’s lunge to hamstring stretch | 10 each side |
| Side lunge walkovers | 8 each side |
| Squat to bilateral straight leg hamstring stretch | 10 reps |
| Ballistic jumps | 5-10-5 seconds (bunny hops, bunny hops for height, tuck jumps) |
| Submaximal HS exercise | 50%, 75%, 85% of max effort for RDLs  50%, 75%, 90% of max effort for NHE |
