# Supplementary figures and images for: The effects of hip- vs. knee-dominant hamstring exercise on biceps femoris morphology, strength, and sprint performance: a randomized intervention trial protocol
Source: BMC Sports Sci Med Rehabil. 2023 Jun 26;15:72. doi: 10.1186/s13102-023-00680-w (PMC10294505; doi:10.1186/s13102-023-00680-w)

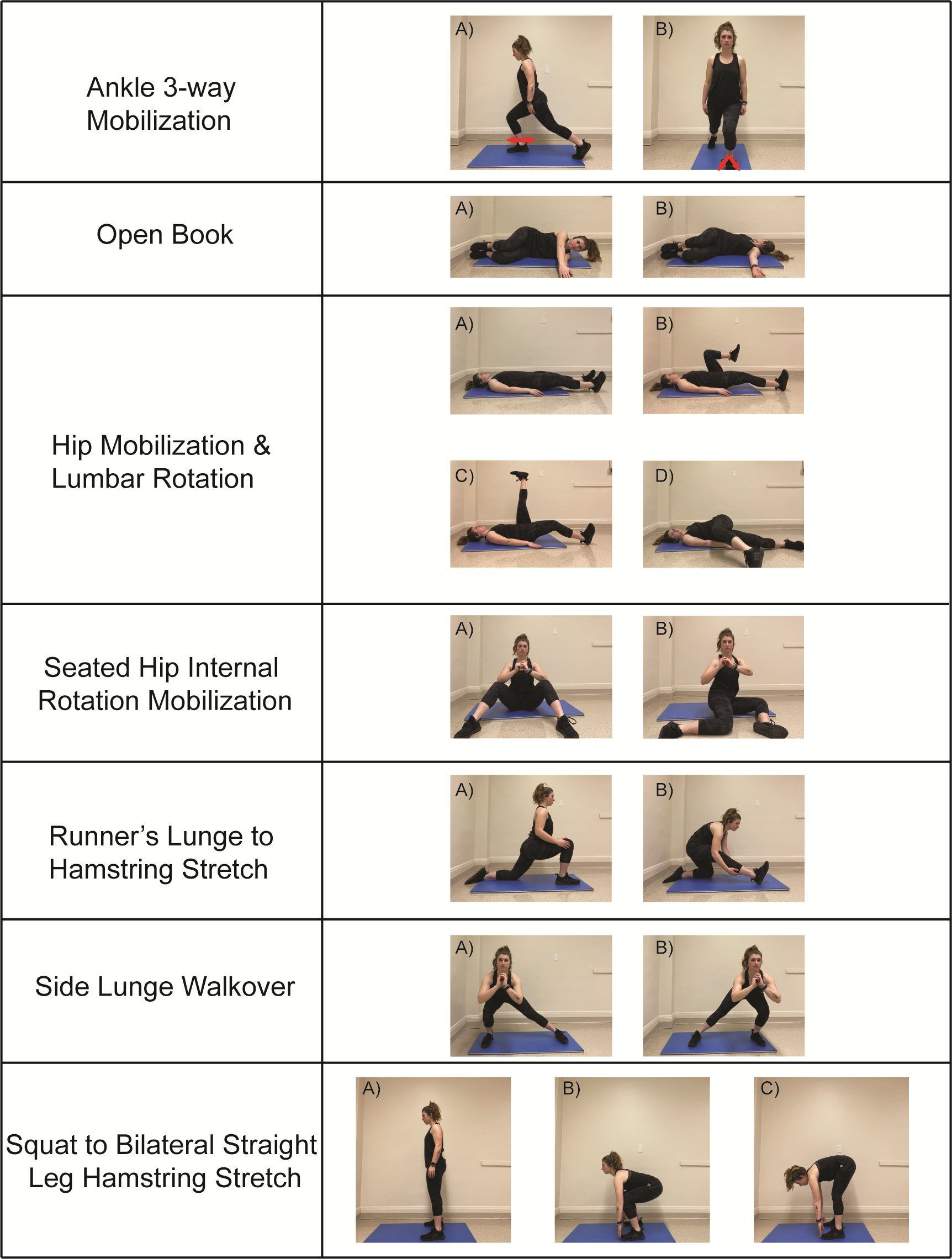

Supplement: Supplementary file 4 — Supplementary Material 4 [file 13102_2023_680_MOESM4_ESM.png]
